# Electrodeposition Stability Landscape for Solid–Solid Interfaces

**DOI:** 10.1002/advs.202307455

**Published:** 2023-12-10

**Authors:** Debanjali Chatterjee, Kaustubh G. Naik, Bairav S. Vishnugopi, Partha P. Mukherjee

**Affiliations:** ^1^ School of Mechanical Engineering Purdue University West Lafayette IN 47907 USA

**Keywords:** Butler–Volmer kinetics, electro‐chemo‐mechanical coupling, electrodeposition stability, solid/solid interface, solid‐state batteries, stack pressure

## Abstract

As solid‐state batteries (SSBs) with lithium (Li) metal anodes gain increasing traction as promising next‐generation energy storage systems, a fundamental understanding of coupled electro‐chemo‐mechanical interactions is essential to design stable solid‐solid interfaces. Notably, uneven electrodeposition at the Li metal/solid electrolyte (SE) interface arising from intrinsic electrochemical and mechanical heterogeneities remains a significant challenge. In this work, the thermodynamic origins of mechanics‐coupled reaction kinetics at the Li/SE interface are investigated and its implications on electrodeposition stability are unveiled. It is established that the mechanics‐driven energetic contribution to the free energy landscape of the Li deposition/dissolution redox reaction has a critical influence on the interface stability. The study presents the competing effects of mechanical and electrical overpotential on the reaction distribution, and demarcates the regimes under which stress interactions can be tailored to enable stable electrodeposition. It is revealed that different degrees of mechanics contribution to the forward (dissolution) and backward (deposition) reaction rates result in widely varying stability regimes, and the mechanics‐coupled kinetics scenario exhibited by the Li/SE interface is shown to depend strongly on the thermodynamic and mechanical properties of the SE. This work highlights the importance of discerning the underpinning nature of electro‐chemo‐mechanical coupling toward achieving stable solid/solid interfaces in SSBs.

## Graphical abstract

This work explores the thermodynamic foundations of mechanics‐coupled reaction kinetics and reveals its implications on electrodeposition stability in solid‐state batteries. Depending on the material properties of the solid electrolyte (SE), lithium (Li) metal/SE interfaces can exhibit distinct mechanics‐driven energetic contributions to the free energy landscape of the reaction, resulting in different scenarios of mechanics‐reaction kinetics coupling and electrodeposition stability regimes.

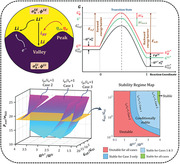

## 1 Introduction

In the face of mounting energy demands and the pressing need to reduce dependence on fossil fuels, solid‐state batteries (SSBs) utilizing a lithium (Li) metal anode have emerged as a promising next‐generation energy storage system.^[^ ^1^ ^]^ Li metal is considered to be the holy grail for unlocking high energy densities, owing to its low reduction potential (−3.04 V) and high specific capacity (3860 mAh/g).^[^ ^2^, ^3^, ^4^ ^]^ Inorganic solid electrolytes (SEs), in theory, can enable compact deposition morphologies and suppress Li dendrites due to their mechanical rigidity.^[^ ^5^, ^6^, ^7^, ^8^ ^]^ With a cationic transference number close to unity, high ionic conductivity, and non‐flammability, they hold important advantages over their liquid counterparts.^[^ ^9^, ^10^, ^11^ ^]^ Several novel strategies such as interlayers^[^ ^12^, ^13^ ^]^ and composite anodes^[^ ^14^ ^]^ have demonstrated notable improvements in the cycle life and rate performance of SSBs.^[^ ^15^, ^16^, ^17^ ^]^

Despite these prospects, realizing the true potential of SSBs is impeded by several fundamental challenges pertaining to the stability of the Li/SE interface.^[^ ^18^, ^19^, ^20^ ^]^ A critical roadblock emerges in the form of unstable interface growth, leading to metal penetration through the SE microstructure and subsequent short circuit.^[^ ^21^, ^22^, ^23^ ^]^ Formation of interfacial voids during stripping has been observed to aggravate this scenario due to current focusing on the contact points.^[^ ^24^, ^25^ ^]^ These interfacial instabilities predominantly stem from the heterogeneous nature of electrochemical reactions at the solid/solid interface. Multiple factors such as the interface morphology,^[^ ^26^ ^]^ SE microstructure (e.g., grain and grain boundary network),^[^ ^27^, ^28^ ^]^ surface defects^[^ ^29^ ^]^ and cathode microstructure^[^ ^30^ ^]^ collectively contribute to the reaction heterogeneity. These intrinsic heterogeneities also lead to non‐uniform stress fields in the SE and Li metal,^[^ ^26^ ^]^ which in turn affect the origin of hotspots and mechanical instability. While creep and viscoplasticity in Li have been shown to play a critical role in replenishing interfacial voids and ensuring contact,^[^ ^31^, ^32^, ^33^ ^]^ they have also been identified as contributing factors to mechanically induced lithium penetration and short circuits.^[^ ^34^, ^35^, ^36^ ^]^

Mechanical stresses at the Li/SE interface are a strong function of the stack pressure, surface morphology, microstructure and mechanical properties of the SE and Li metal.^[^ ^33^, ^37^ ^]^ Previous studies have revealed the role of interfacial stresses in influencing reaction kinetics and connected it to electrodeposition stability. For example, Monroe and Newman^[^ ^38^, ^39^ ^]^ theorized a direct correlation between mechanical stresses and electrochemical potential. Considering Li metal and the SE to be linearly elastic, their findings indicated that the shear modulus of the SE should be at least twice that of Li in order to suppress the growth of dendritic morphologies. This analysis was extended by Barai et.al. by incorporating the plasticity of Li metal.^[^ ^40^ ^]^ Furthermore, the role of molar volume ratio ($\Omega^{Li^{+}}/\Omega^{Li}$) on electrodeposition stability has been investigated, suggesting that a combination of high (low) molar volume ratio and high (low) SE shear modulus is necessary for stable morphological growth.^[^ ^41^ ^]^ Additionally, a recent study proposed that mechanical stresses can alter not only reaction kinetics but also Li^+^ transport through the SE, and the competing effects of stress‐driven reaction kinetics and stress‐driven transport dictate the interface stability.^[^ ^42^ ^]^ On the experimental front, a recent study examined the electro‐chemo‐mechanical behavior of Li metal combined with a garnet electrolyte by measuring the stack pressure‐dependent resistance at the Li/SE interface.^[^ ^43^ ^]^ Another experimental investigation explored the impact of mechanical stress states on the equilibrium potential in alkali metal‐SE systems.^[^ ^44^ ^]^

Despite significant progress, a deeper understanding of the role of mechanics in dictating reaction kinetics at the Li/SE interface is still required. In particular, studies across literature have considered different mechanics‐coupled Butler‐Volmer formulations to capture the reaction kinetics at the solid/solid interface.^[^ ^45^ ^]^ The disparity in these formulations is linked to the differences in the underlying assumptions about the mechanics contribution to the forward (dissolution) and backward (deposition) reactions. For example, some studies have proposed that mechanical stresses affect both the forward and backward reaction rates,^[^ ^46^ ^]^ while others have assumed that stresses selectively impact either the forward^[^ ^39^, ^42^ ^]^ or the backward reaction rate.^[^ ^47^ ^]^ Herein, we hypothesize that the stability landscape for Li metal electrodes in SSBs is strongly dictated by how mechanical stresses alter the energetic states of the interacting species and influence reaction kinetics. In this context, a comprehensive understanding of the thermodynamic origins of the role of mechanics in reaction kinetics and its implications on electrodeposition stability is necessary. In addition, it is important to mechanistically interrogate how instabilities scale with intrinsic (e.g., interface morphology and molar volume ratio) and extrinsic (e.g., stack pressure, temperature, and current density) factors for solid/solid interfaces exhibiting distinct mechanics‐reaction kinetics interactions.

In this work, we investigate the thermodynamic origin of the role of mechanics on reaction kinetics and reveal its implications on electrodeposition stability in SSBs. Through a bottom‐up approach that builds on the transition state theory, we determine how mechanical stresses alter the free energy landscape of the redox reaction at the Li/SE interface and correlate it to changes in the equilibrium potential and exchange current density. Cognizant of the mechanics contribution to the forward (dissolution) and backward (deposition) reaction rates, we present different mechanics‐coupled Butler‐Volmer kinetics formulations and develop an electro‐chemo‐mechanical modeling framework that systematically connects our thermodynamic description to the interface stability during plating. Through this analysis, we examine the individual roles of mechanical and electrical overpotentials in dictating the reaction heterogeneity and establish that their variation along the interface acts as the driving force for electrodeposition stability. Moreover, we show that the competing effects of mechanical stresses and electric potential are strongly influenced by intrinsic factors such as molar volume ratio and shear moduli, as well as extrinsic factors such as interface morphology (surface roughness), stack pressure, applied current density and operating temperature. We reveal that distinct natures of mechanics‐reaction kinetics coupling can give rise to widely disparate electrodeposition stability regimes. This study sheds light on the importance of identifying how mechanics contributes to reaction kinetics and delineates design guidelines toward achieving stable electrodeposition in SSBs.

## 2 Thermodynamic Analysis Using Transition State Theory

**Figure 1:**
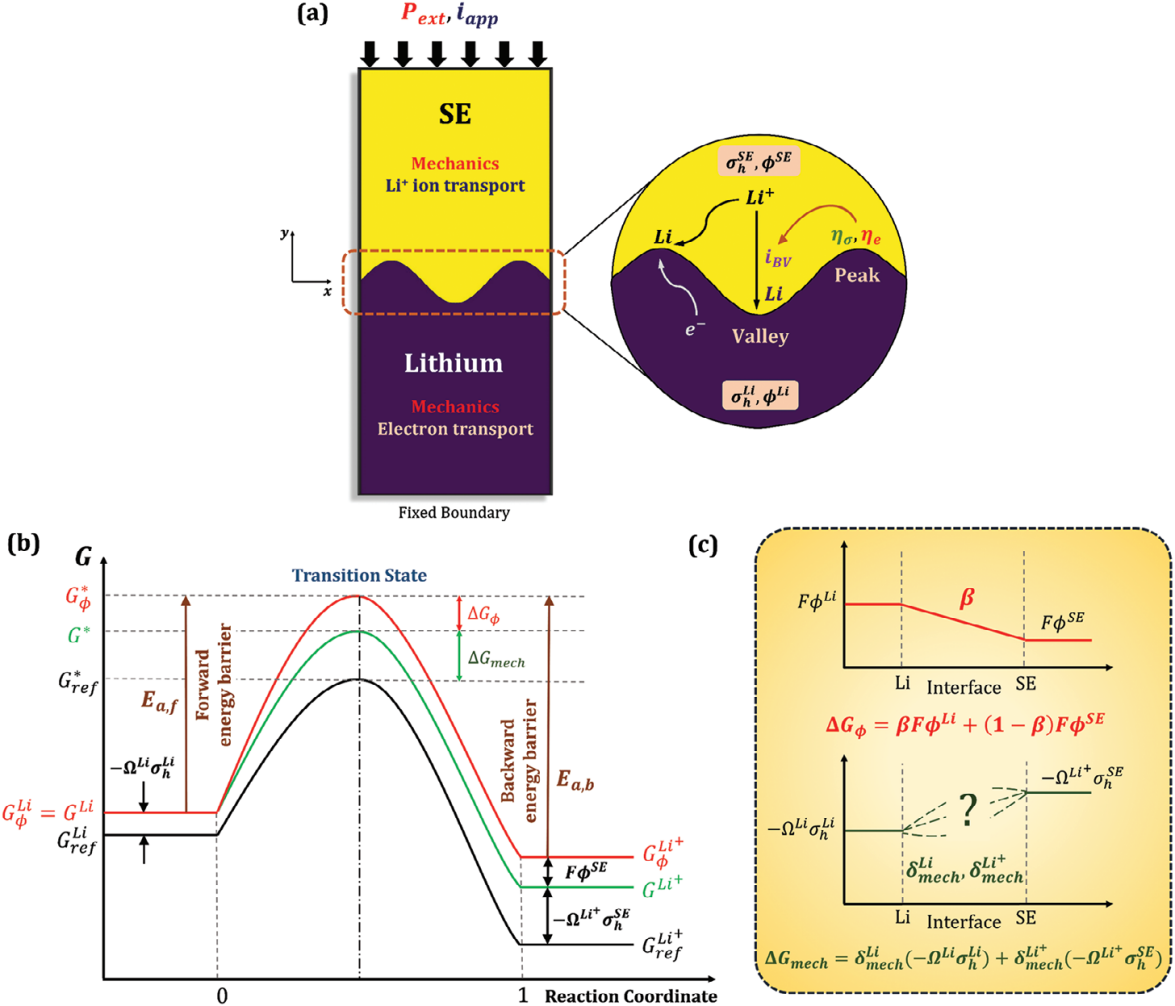
a) Schematic illustration of the model domain consisting of Li metal electrode and the SE. Electrodeposition kinetics at the Li/SE interface is dictated by coupled electrochemical‐mechanical interactions. b) Energy landscape of the redox reaction occurring at the Li/SE interface. c) Both mechanical stresses and electric fields alter the free energies of the Li and Li^+^ ion, which serves as the fundamental basis for the role of mechanics in reaction kinetics.

**Figure** 1a presents a schematic illustration of the model domain considered in this work. Li^+^ transport in the SE, electron transport in Li metal, reaction kinetics at the Li/SE interface and mechanical stresses within the domain collectively govern the Li deposition behavior at the interface. The electrochemical redox reaction at the interface can be represented as follows:

(1) $$Li⇌Li^{+}+e^{-}$$

With the electrodeposition of fresh metal, stresses arising due to mechanical constraints at the solid/solid interface can contribute to the overall free energy of the redox reaction, affecting electrodeposition kinetics. This warrants a deep dive into the effect of mechanics on the reaction kinetics, cognizant of the fundamental thermodynamic interactions via the transition‐state theory. Figure 1b depicts the energy landscape of the redox reaction at the Li/SE interface. In the absence of any electric potential, the energetic states of Li atom in the metal electrode and Li^+^ ion in the SE are specified by their Gibbs free energies, $G^{\mathrm{Li}}$ and $G^{Li^{+}}$ respectively, and can be expressed as follows:

(2) $$G^{Li}=G_{ref}^{Li}\;-\Omega^{Li}\sigma_{h}^{Li}$$

(3) $$G^{Li^{+}}=G_{ref}^{Li^{+}}\;-\Omega^{Li^{+}}\sigma_{h}^{SE}$$

where $G_{\mathrm{ref}}$ denotes the reference value of the Gibbs free energy and $G_{\mathrm{mech}}=-\Omega \sigma_{h}$ denotes the contribution from the mechanical stresses. Here, $\Omega^{\mathrm{Li}}$ is the molar volume of Li metal, $\Omega^{Li^{+}}$ is the partial molar volume of Li^+^ ion in the SE, and $\sigma_{h}^{Li}$ and $\sigma_{h}^{SE}$ are the interfacial hydrostatic stresses in Li metal and the SE, respectively. It is noted that the free energy contribution arising due to the concentration difference, $G_{conf}=\;RT\ln\left(\frac{c}{c_{ref}}\right)$, is considered to be much smaller due to the negligible concentration gradients in the SE ($c=c_{\mathrm{ref}}$).^[^ ^48^ ^]^

We now look at the progress of the electrochemical reaction by considering the path it takes along the energy landscape. The highest point in the energy landscape corresponds to the energy of the transition state, $G^{*}$. Mathematically, $G^{*}$ can be represented as follows:

(4) $$G^{*}=G_{ref}^{*}\;+\delta_{mech}^{Li}G_{mech}^{Li}+\delta_{mech}^{Li^{+}}G_{mech}^{Li^{+}}$$

where $\delta_{mech}^{Li}$ and $\delta_{mech}^{Li^{+}}$ are mechanics coefficients which account for the dependence of the barrier energy on the energies of the Li atom in the anode and Li^+^ ion in the SE (Figure 1c). Here, each energy term corresponding to the Li atom and Li^+^ ion can either contribute fully, partially, or not contribute at all to the transition state energy. Thus, each mechanics coefficient must satisfy: $0\leq \delta_{\mathrm{mech}}\leq 1$. For example, if $\delta_{mech}^{Li^{+}}=\;1$ and $\delta_{mech}^{Li}=\;0$, then the transition state energy $G^{*}$ depends only on the energetic state of the Li^+^ion. Similarly, $\delta_{mech}^{Li^{+}}=\;0$ and $\delta_{mech}^{Li}=\;1$ indicate that $G^{*}$depends only on the energetic contribution of stresses in Li metal.

We now introduce the effect of electric potential on the energy landscape. Considering electric potentials $\phi^{\mathrm{Li}}$ and $\phi^{\mathrm{SE}}$ of Li metal and the SE, respectively, the energetic states of Li atom and Li^+^ ion are altered as follows:

(5) $$G_{\phi }^{Li}=G^{Li}\;+F\phi^{Li}$$

(6) $$G_{\phi }^{Li^{+}}=G^{Li^{+}}\;+F\phi^{SE}$$

Here, $G_{\phi }^{Li}$ and $G_{\phi }^{Li^{+}}$ denote the energetic states of Li atom and Li^+^ ion in the presence of electric potentials $\phi^{\mathrm{Li}}$ and $\phi^{\mathrm{SE}}$, respectively, and *F* is the Faraday constant. Since the variation in electric potential should be continuous across Li metal and the SE, we consider that it changes linearly along the reaction coordinates as depicted in Figure 1c. Thus, the transition state energy, $G_{\phi }^{*}$, becomes:

(7) $$G_{\phi }^{*}=G^{*}\;+F\left[\beta \phi^{Li}+\left(1-\beta \right)\phi^{SE}\right]$$

Here β is the symmetry factor which determines the position of the transition state energy $G_{\phi }^{*}$ along the reaction coordinate. We note that previous studies have indicated β=0.5 as a suitable value for the symmetry factor.^[^ ^49^, ^50^ ^]^

With this framework established, the activation energy barriers of the forward and backward reactions can be evaluated as $E_{a,f}=G_{\phi }^{*}\;-G_{\phi }^{Li}$ and $E_{a,b}=G_{\phi }^{*}\;-G_{\phi }^{Li^{+}}$, and the corresponding reaction rates as $r_{f}=\frac{k_{B}T}{h}\;\exp\left(-\frac{E_{a,f}}{RT}\right)$ and $r_{b}=\frac{k_{B}T}{h}\;\exp\left(-\frac{E_{a,b}}{RT}\right)$, respectively. Here, $k_{B}$ is the Boltzmann constant, *h* is the Planck's constant, *R* is the universal gas constant and *T* is the temperature. The current densities associated with the forward and backward reactions are: $i_{f}=Fc_{s}r_{f}$ and $i_{b}=Fc_{s}r_{b}$, where $c_{s}$ is the surface concentration of Li atoms at the interface. This can be further expanded as follows:

(8) $$\begin{array}{ccc} i_{f} & = & K\exp\left(-\frac{\left(G_{ref}^{*}-G_{ref}^{Li}\right)+\left(\delta_{mech}^{Li}-1\right)G_{mech}^{Li}+\delta_{mech}^{Li^{+}}G_{mech}^{Li^{+}}}{RT}\right)\\ & & \times \;\exp\left(\frac{\left(1-\beta \right)F\left(\phi^{Li}-\phi^{SE}\right)}{RT}\right) \end{array}$$

(9) $$\begin{array}{ccc} i_{b} & = & K\exp\left(-\frac{\left(G_{ref}^{*}-G_{ref}^{Li^{+}}\right)+\;\delta_{mech}^{Li}G_{mech}^{Li}+\left(\delta_{mech}^{Li^{+}}-1\right)G_{mech}^{Li^{+}}}{RT}\right)\\ & & \times \;\exp\left(-\frac{\beta F\left(\phi^{Li}-\phi^{SE}\right)}{RT}\right) \end{array}$$

where $K\;=\frac{k_{B}T}{h}\;Fc_{s}$. In equilibrium, the forward and backward current densities are equal, therefore, the net current is zero. The potential difference $\phi^{\mathrm{Li}}-\phi^{\mathrm{SE}}$ in this case is termed as the equilibrium potential, *U*, and the corresponding forward/backward current is the exchange current density, *i*_0_. Equating the forward and backward reaction currents, we get the expression for the equilibrium potential as follows:

(10) $$U\;=\;\frac{-\Delta G_{ref}}{F}-\;\frac{\Omega^{Li^{+}}\sigma_{h}^{SE}-\Omega^{Li}\sigma_{h}^{Li}}{F}=E^{0}\;-\eta_{\sigma }$$

Where $\Delta \;G_{ref}=G_{ref}^{Li}\;-G_{ref}^{Li^{+}}$ is the Gibbs free energy change of the Li deposition reaction $Li^{+}+e^{-}\to \mathrm{Li}$, and $E^{0}=-\Delta G_{\mathrm{ref}}/F$ is the equilibrium potential of the same reaction at standard temperature and pressure, respectively. The additional term $\eta_{\sigma }$ is the change in the equilibrium potential brought about by mechanical stresses and is termed as the mechanical overpotential. Here, we note that the equilibrium potential, *U*, is independent of the mechanics coefficients $\delta_{mech}^{Li}$ and $\delta_{mech}^{Li^{+}}$ which govern the selective influence of mechanics on the forward/backward reactions.

Substituting Equation (10) in Equations (8) and (9), we get the mechanics‐incorporated exchange current density as:

(11) $$\begin{array}{c} i_{0}=i_{00}\;\exp\left(-\frac{\left(\beta -\delta_{mech}^{Li}\right)\Omega^{Li}\sigma_{h}^{Li}+\left(1-\beta -\delta_{mech}^{Li^{+}}\right)\Omega^{Li^{+}}\sigma_{h}^{SE}\;}{RT}\right) \end{array}$$

where, *i*_00_ is a constant dependent on the reference values of the free energies. We henceforth refer to this as the mechanics‐independent exchange current density.

Subsequently, we look at the non‐equilibrium case where the forward and backward reaction currents are unequal. The net current density, $i=i_{f}-i_{b}$, can be expanded and expressed as follows:

(12) $$\begin{array}{ccc} i & = & i_{0}\;\left[\exp\left(\frac{\left(1-\beta \right)F\eta }{RT}\right)-\exp\left(\frac{-\beta F\eta }{RT}\right)\right],\\ \eta & = & \phi^{Li}-\phi^{SE}-U \end{array}$$

Here, the overpotential, η, can be further expanded from Equation (10) as:

(13) $$\eta \;=\left(\phi^{Li}-\phi^{SE}-E^{0}\right)\;+\;\eta_{\sigma }=\;\eta_{e}+\eta_{\sigma }$$

Thus, the total overpotential is the sum of electrical and mechanical overpotentials.

In order to understand the role of mechanics on exchange current density (Equation (11)), we take a closer look at the mechanics coefficients $\delta_{mech}^{Li}$ and $\delta_{mech}^{Li^{+}}$. These coefficients are descriptors of the selective effect of mechanics on the energy barriers of the forward/backward reaction. For example, if $\delta_{mech}^{Li}=\;0$ and $\delta_{mech}^{Li^{+}}=\;1$, the energy barriers become:

(14) $$E_{a,f}=G_{ref}^{*}\;-G_{ref}^{Li}+\left(G_{mech}^{Li^{+}}-G_{mech}^{Li}\right)$$

(15) $$E_{a,b}=G_{ref}^{*}\;-G_{ref}^{Li^{+}}$$

This implies that mechanics selectively contributes to the energy barrier of the forward reaction, whereas the backward reaction energy barrier is independent of any mechanics contribution. A similar analysis for $\delta_{mech}^{Li}=\;1$ and $\delta_{mech}^{Li^{+}}=\;0$ indicates a case where mechanical stresses selectively contribute to the energy barrier of the backward reaction only. Based on the nature of this selectivity, different mechanics‐coupled kinetic formulations are obtained, as listed below in **Table** **1**.

**Table 1:** Mechanics‐coupled Butler‐Volmer kinetics formulations for different mechanics‐driven energetic contributions to reaction kinetics at the Li/SE interface.

Case	Mechanics‐driven energetic contribution to reaction kinetics	$\delta_{mech}^{Li}$	$\delta_{mech}^{Li^{+}}$	*i*_0_	*i_BV_*
1	Equally to forward and backward reaction rates	0.5	0.5	*i*_00_	$i_{00}\left[\exp\left(\frac{F(\eta_{\sigma }+\eta_{e})}{2RT}\right)-\exp\left(-\frac{F(\eta_{\sigma }+\eta_{e})}{2RT}\right)\right]$
2	Only to forward reaction rate	0	1	$i_{00}\exp\left(\frac{F\eta_{\sigma }}{2RT}\right)$	$i_{00}\left[\exp\left(\frac{F\eta_{\sigma }}{RT}\right)\exp\left(\frac{F\eta_{e}}{2RT}\right)-\exp\left(-\frac{F\eta_{e}}{2RT}\right)\right]$
3	Only to backward reaction rate	1	0	$i_{00}\exp\left(-\frac{F\eta_{\sigma }}{2RT}\right)$	$i_{00}\left[\exp\left(\frac{F\eta_{e}}{2RT}\right)-\exp\left(\frac{-F\eta_{\sigma }}{RT}\right)\exp\left(-\frac{F\eta_{e}}{2RT}\right)\right]$

Cases 2 and 3 represent limiting scenarios of mechanics‐driven contributions to the free energy landscape of the reaction. In Case 2, the transition state free energy depends solely on mechanical stresses within the SE, while in Case 3, it relies exclusively on the mechanical stresses within Li metal. Case 1 falls between these two scenarios, with equal contributions from Li^+^ ion (in the SE) and Li metal. Consequently, our analysis covers the entire spectrum of mechanics‐driven contributions from the reacting species to the forward and backward reaction rates.

This analysis is incorporated in the coupled electro‐chemo‐mechanical model to study the role of mechanics in reaction kinetics and systematically connected to the electrodeposition stability at the Li/SE interface. A detailed description of the modeling framework and the parameters used in this study are presented in Section S1, Tables S1 and S2 (Supporting Information).

## 3 Results and Discussion

**Figure 2:**
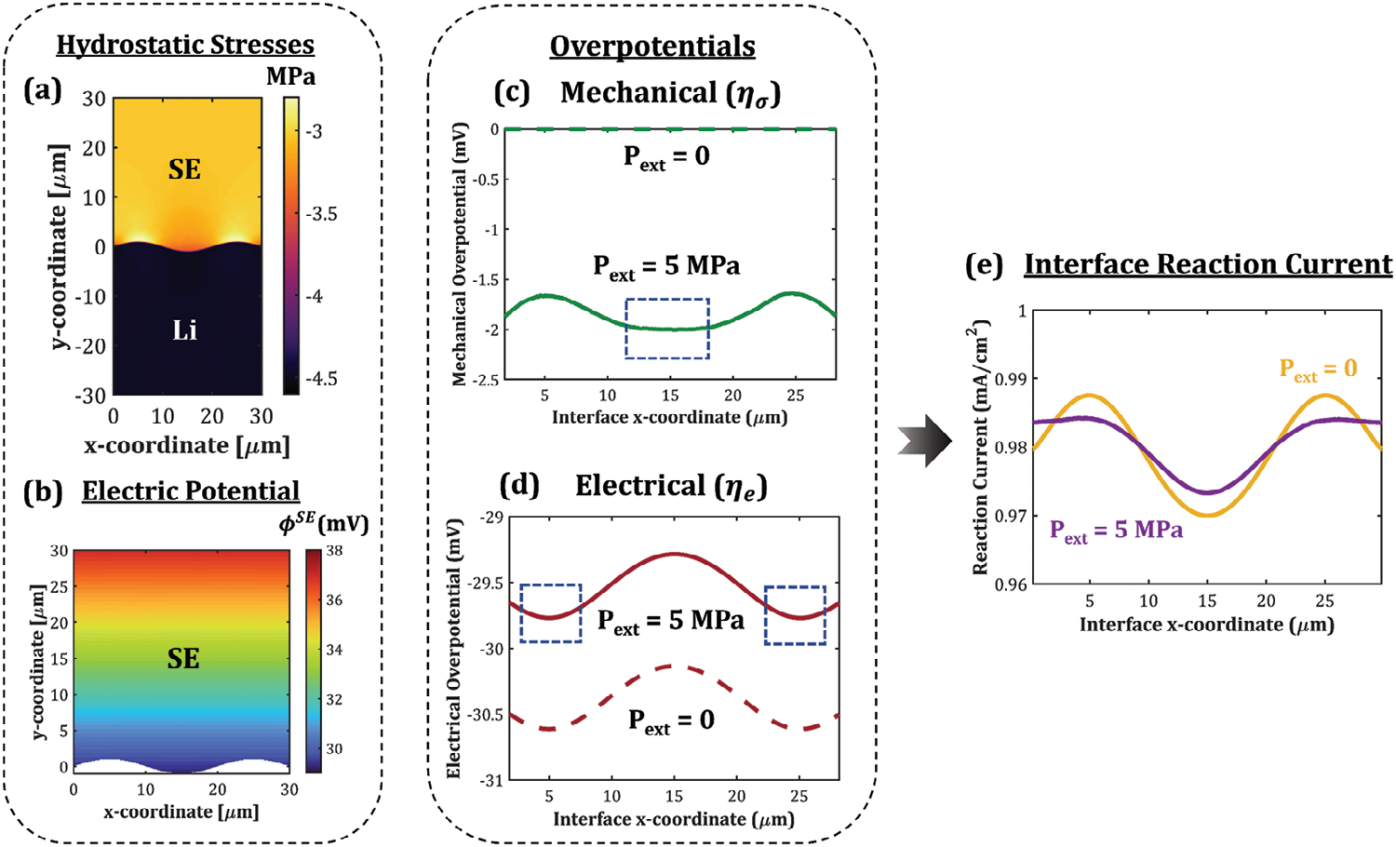
Effect of mechanical stresses on reaction distribution at the Li/SE interface. a) Hydrostatic stresses in Li metal and SE for a stack pressure of 5 MPa. b) Electric potential in the SE domain for applied current density of 1 mA cm^−2^ and stack pressure of 5 MPa. c) Mechanical and d) electrical overpotentials at the Li‐SE interface for 0 and 5 MPa stack pressures. e) Reaction distribution at the Li/SE interface for 0 and 5 MPa stack pressures at 1 mA cm^−2^ current density.

**Figure 3:**
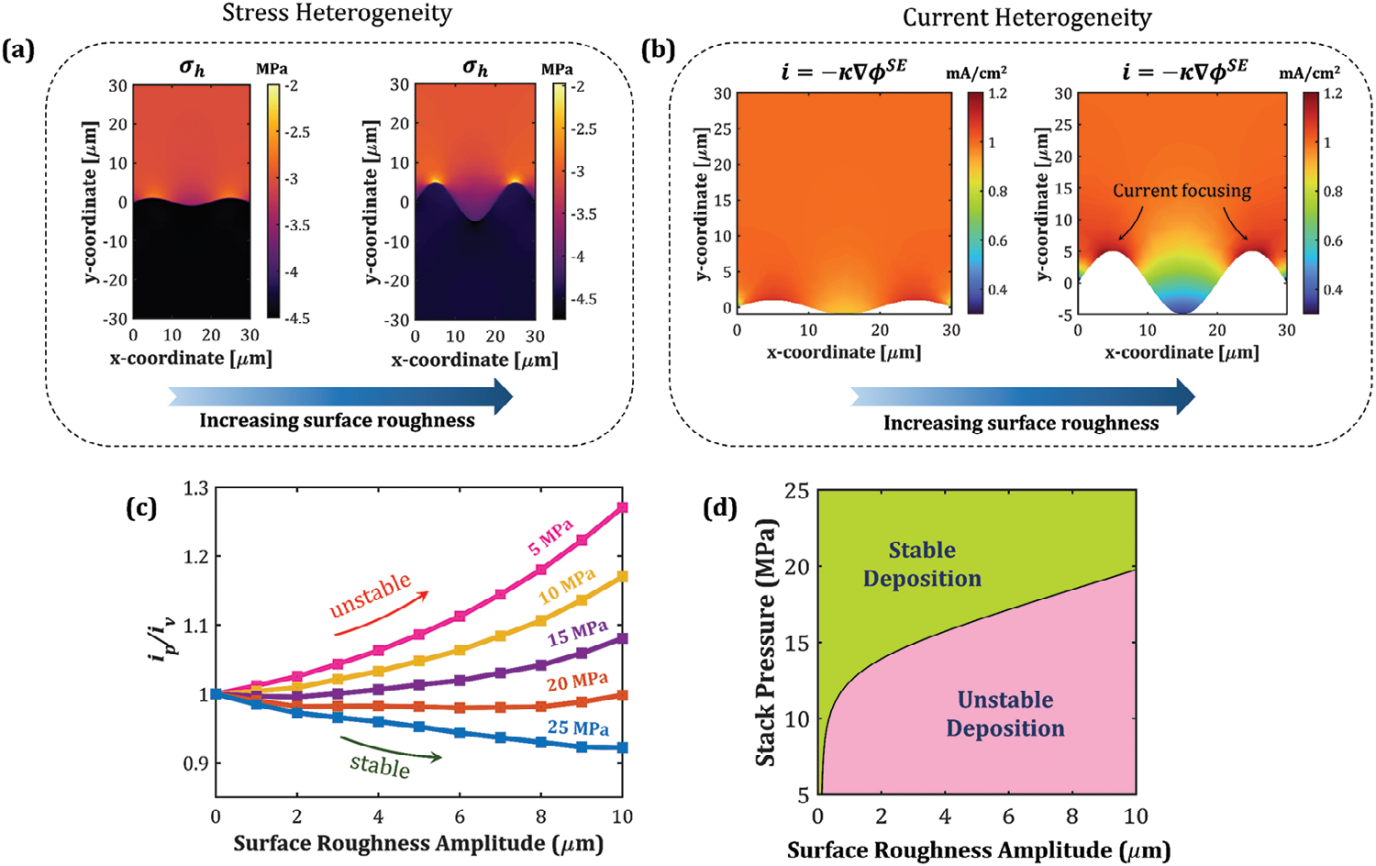
Effect of interface morphology on electrodeposition stability. a) Hydrostatic stresses for a stack pressure of 5 MPa and b) current distribution in the SE, illustrating the reaction focusing effect on the interface peaks. c) Stability descriptor, $i_{p}/i_{v}$, as a function of the surface roughness amplitude for different stack pressures. d) Stability regime map as a function of surface roughness amplitude and stack pressures.

**Figure 4:**
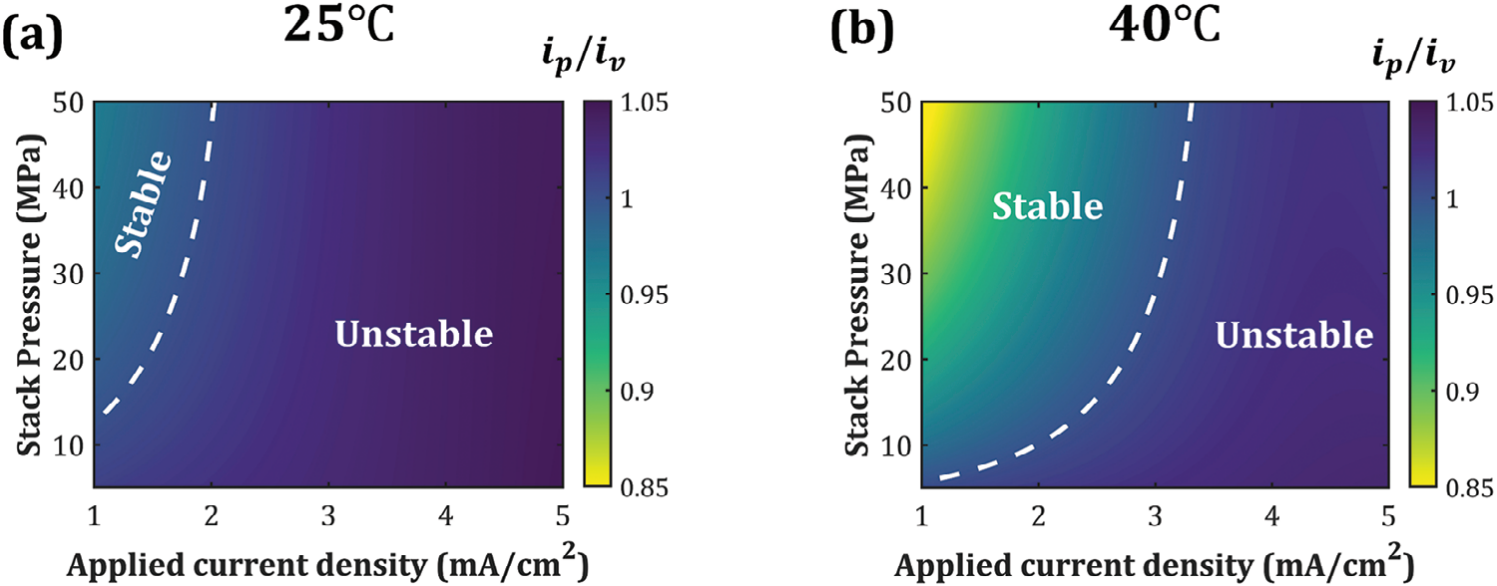
Effect of temperature on Li deposition stability at the Li/SE interface. a,b) Electrodeposition stability descriptor, $i_{p}/i_{v}$, as a function of stack pressure and applied current density for an operating temperature of a) 25 °C and b) 40 °C, respectively. Here, the surface roughness amplitude is kept constant at 1 µm. The white dashed line represents the contour $i_{p}/i_{v}=1$.

As established using the transition state theory, mechanical stresses and electric potential jointly affect the reaction kinetics at the Li/SE interface. Building on this thermodynamic basis, we investigate the electrodeposition behavior under a scenario where mechanical stresses affect only the rate of the forward (dissolution) reaction (Case 2, **Table** **1**) and present the results in **Figures** 2, 3, 4. Figure 2 compares the reaction distribution at the interface for stack pressures of 0 and 5 MPa, and an applied current density of 1 mA cm^−2^. Figure 2a presents the hydrostatic stress distribution within the Li metal and SE for a stack pressure of 5 MPa, and Figure 2b represents the corresponding electric potential distribution in the SE. Here, due to its low yield strength (0.8 MPa),^[^ ^51^ ^]^ Li metal undergoes plastic deformation and shows negligible variation in the hydrostatic stresses. On the other hand, the hydrostatic stresses in the SE show significant variation along the interface. In particular, hydrostatic stresses at the interface are more compressive at the valleys than at the peaks. Such non‐uniform stresses result in a heterogeneous distribution of mechanical overpotential at the interface, as shown in Figure 2c. Thermodynamically, Li^+^ ions will be preferably reduced to Li metal at those interfacial locations where hydrostatic stresses in the SE are more compressive, i.e., mechanical overpotential is more negative (Equation (10)). Therefore, mechanical stresses direct Li deposition at the interface valleys and have a stabilizing effect. On the contrary, owing to the higher electric potential gradients (Figure 2b), the electrical overpotential (Figure 2d) drives preferential Li deposition at the peaks. Thus, the electric potential favors unstable electrodeposition at the interface via current focusing on the peaks. Overall, the reaction distribution and electrodeposition stability at the interface is determined by two competing effects – mechanics‐driven stabilization and electric potential‐driven destabilization. As shown in Figure 2e, the interfacial reaction distribution under an applied pressure of 5 MPa is more stable as compared to a pressure‐free scenario.

The mechanical stress distribution and electric potential gradients in the interface vicinity are a strong function of interface morphology. To investigate its effect on electrodeposition stability, we model the interface as a sinusoidal function y=Asin(ωx), where *A* and ω denote the surface roughness amplitude and frequency, respectively. Increasing surface roughness amplifies the heterogeneity in both mechanical stresses and electric potential gradient at the interface, as depicted in Figure 3a,b, respectively. While the heterogeneity in electric potential gradient leads to current focusing on the interface peaks, the heterogeneity in interfacial stresses drives more current into the valleys. Thus, interface morphology governs both the destabilizing and stabilizing mechanisms, and has a crucial impact on the electrodeposition stability.

We define the ratio of reaction current at the peak to that at the valley, $i_{p}/i_{v}$, as a descriptor of the electrodeposition stability. Figure 3d shows $i_{p}/i_{v}$ as a function of the surface roughness amplitude, *A*, for stack pressures ranging from 5 to 25 MPa. For a stack pressure of 5 MPa,$i_{p}/i_{v}$ is always greater than 1 and increases monotonically with surface roughness, implying unstable electrodeposition for all surface roughness values. This can be explained by comparing the contributing effects of mechanical stresses and electric potential on the interfacial reaction kinetics. At 5 MPa, the mechanics‐assisted stabilization is not enough to negate the destabilizing influence of the electric potential distribution; this destabilizing effect exacerbates with an increase in surface roughness (Figure 3d). A similar trend is observed at a stack pressure of 10 MPa. However, as stack pressure is increased further, the mechanistic interactions are starkly altered. For a stack pressure of 15 MPa, $i_{p}/i_{v}$ shows a slight decrease up to a surface roughness amplitude of 2 µm, and then rises with further increase in surface roughness. In fact, stable electrodeposition ($i_{p}/i_{v}<1$) is achieved up to an amplitude of 3 µm. A similar trend is observed for 20 MPa stack pressure, where $i_{p}/i_{v}$ decreases up to an amplitude of 6 µm and stable electrodeposition is obtained up to an amplitude of 10 µm (Figure 3d). For these cases, as surface roughness is increased, the mechanics‐assisted stabilization compensates for the electric potential‐driven destabilization. With further increase in the surface roughness, the destabilizing effect dominates the stabilizing effect, resulting in unstable electrodeposition. This indicates that the mechanical overpotential and electric overpotential show dissimilar trends with variation in the surface roughness, and their competing effects dictate the electrodeposition stability. For higher stack pressures (i.e., > 25 MPa), $i_{p}/i_{v}$ shows a continuous decrease with increasing surface roughness, implying a stable electrodeposition regime. Here, the mechanics‐driven stabilization dominates over the destabilizing effect of electric potential across the entire range of surface roughness values considered here (0–10 µm).

As the difference in the mechanical overpotential between the peak and the valley increases, mechanics‐driven stabilization is enhanced. Analogously, the larger the difference in the electrical overpotential between the peak and the valley, greater is the electric potential‐driven destabilization. Thus, the difference in mechanical/electrical overpotentials between the peak and the valley acts as a critical driving force for interfacial instability. For example, Figure S2a (Supporting Information) explains the trend observed for the 5 MPa stack pressure case. Here, the electrical overpotential difference between the peak and the valley is greater than the corresponding mechanical overpotential difference, implying that electric potential‐driven destabilization dominates over mechanics‐assisted stabilization for all surface roughness values. On the other hand, at 25 MPa stack pressure (Figure S2b, Supporting Information), the mechanical overpotential difference is always greater than the electrical overpotential counterpart, rendering a stable electrodeposition response for all the interface morphologies considered. Figure 3e depicts the corresponding stability regime map which suggests that if the surface roughness of the Li/SE interface is known, the applied stack pressure can be tuned in such a way that stable electrodeposition can be achieved.

Toward achieving stable electrodeposition at low stack pressures and higher charging rates, we explore the role of operating temperature in Figure 4. Figure 4a,b depict the stability descriptor, $i_{p}/i_{v}$, as a function of the stack pressure and applied current density for operating temperatures of 25 and 40 °C, respectively. For this analysis, the surface roughness amplitude is kept constant at 1 µm. Increasing the applied current density, $i_{\mathrm{app}}$, intensifies the heterogeneity in electric potential gradients along the interface, which favors unstable electrodeposition due to current focusing on the peaks. On the other hand, increasing stack pressure can enable stable electrodeposition via mechanics‐driven stabilization. Now, increasing the operating temperature from 25 to 40°C broadens the stability regime as demarcated in Figure 4a,b. For example, at 25 °C, applying a stack pressure of 20 MPa allows stable electrodeposition ($i_{p}/i_{v}<1$) up to $i_{\mathrm{app}}$ = 1.44 mA cm^−2^. Whereas at 40 °C, stable electrodeposition can be achieved with applied current density as high as 2.74 mA cm^−2^ for the same stack pressure. A rise in temperature results in improved Li^+^ ion transport and elevated reaction rate owing to an increase in the ionic conductivity and exchange current density, respectively (Equations S7 and S8, Supporting Information). The broader stability zone at higher temperature is attributed to the efficient ionic transport, which ensures uniform electric potential gradients near the interface, reducing the destabilizing effect of electric potential and enabling stable electrodeposition. Thus, understanding the mechanistic co‐dependence of electrodeposition stability on stack pressure and temperature is critical toward designing stable Li/SE interfaces, especially for faster charging rates.

**Figure 5:**
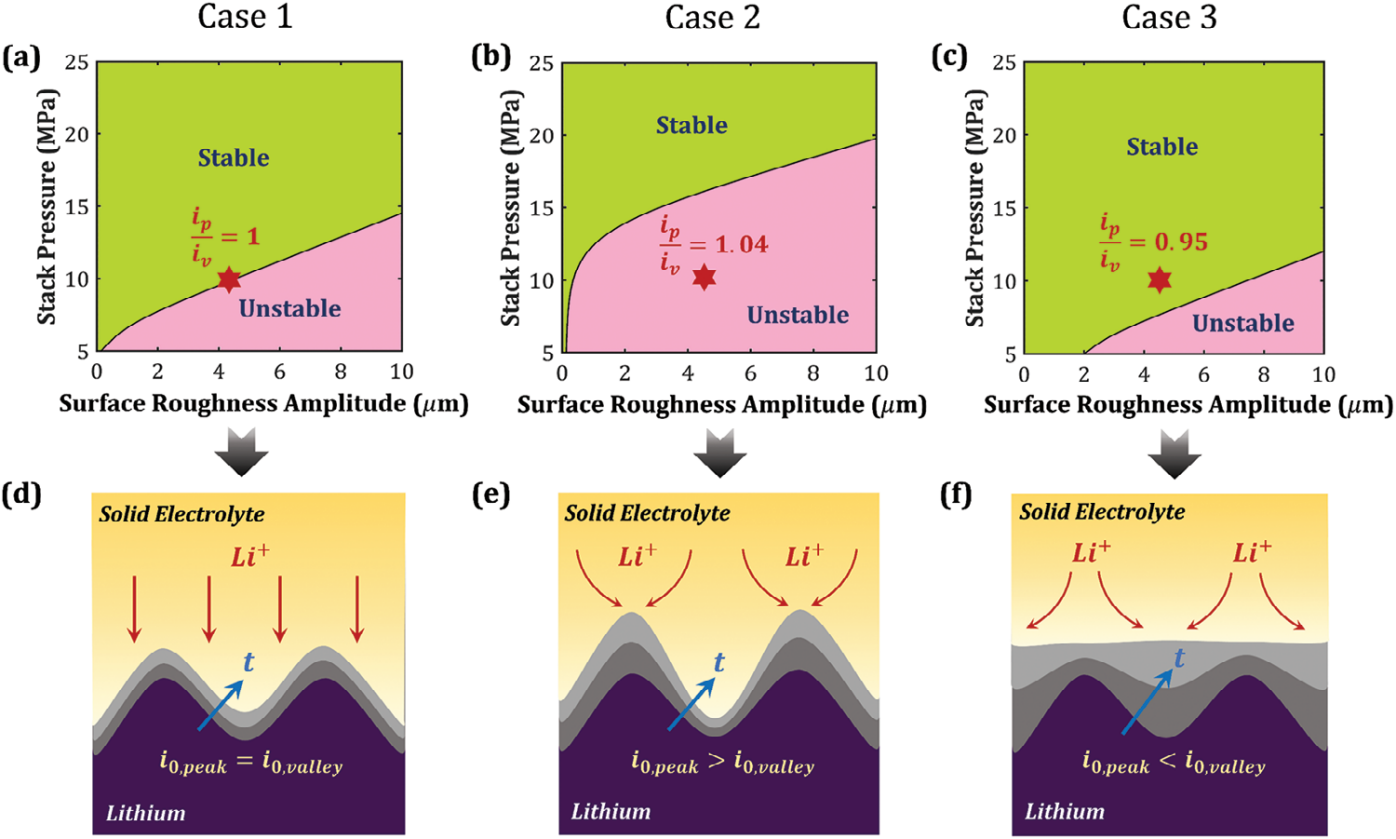
Electrodeposition stability as a function of stack pressure and surface roughness amplitude for three mechanics‐coupled reaction kinetics formulations listed in Table 1: a) Case 1, where mechanics affects the forward and backward reaction rates equally; b) Case 2, where mechanics affects only the forward reaction rate and c) Case 3, where mechanics affects only the backward reaction rate. Schematics depict how mechanics‐dependent exchange current density influences electrodeposition stability for d) Case 1, e) Case 2, and f) Case 3. Results shown here are for an applied current density of 1 mA cm^−2^.

**Figure 6:**
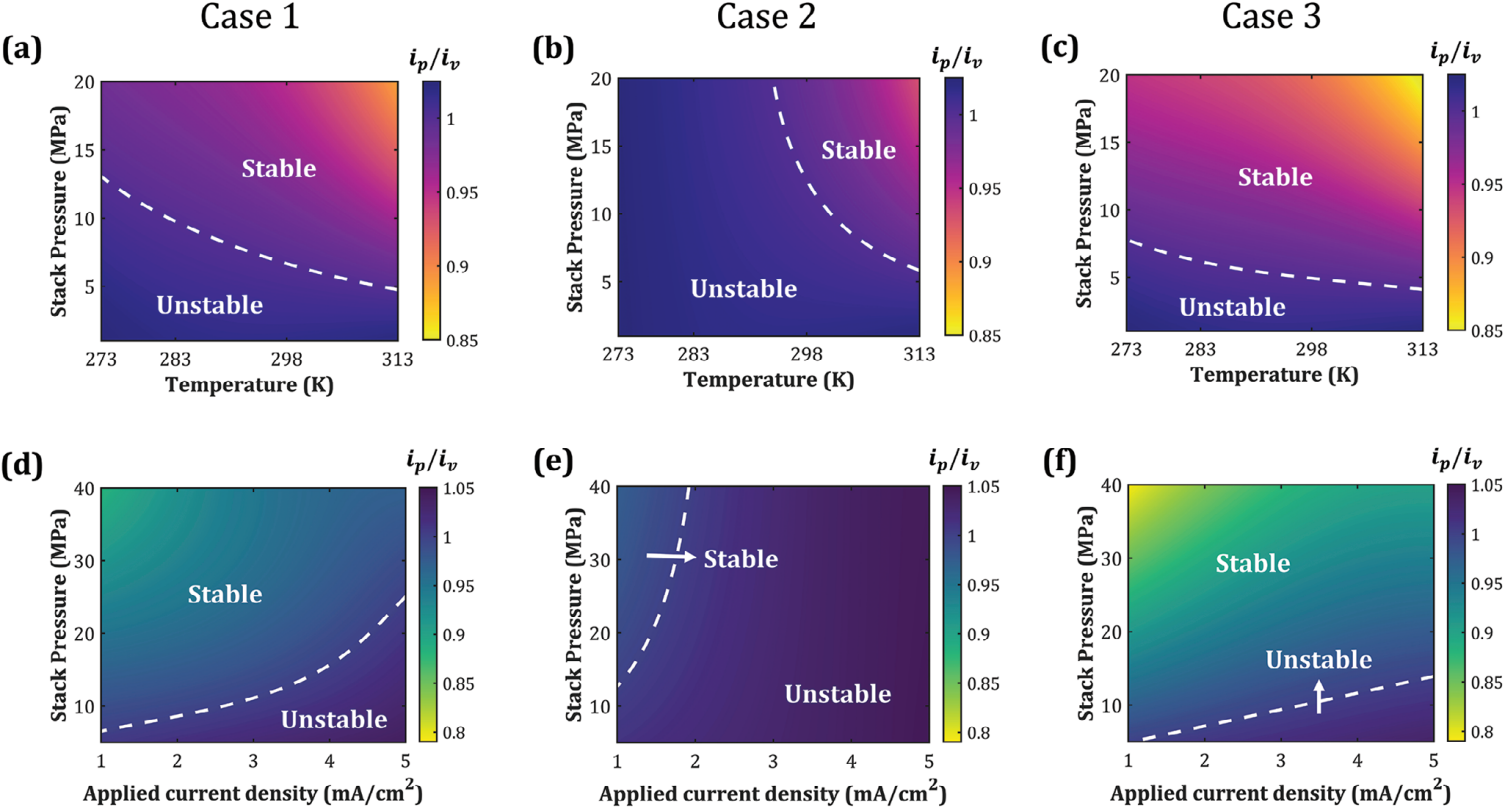
Electrodeposition stability as a function of stack pressure and operating temperature for three mechanics‐coupled reaction kinetics formulations listed in Table 1: a) Case 1; b) Case 2; and c) Case 3. Stability regimes as a function of stack pressure and applied current density for: d) Case 1; e) Case 2; and f) Case 3.

Depending on the SE material, Li/SE interfaces can potentially exhibit differing degrees of mechanics‐reaction kinetics coupling; this interaction is primarily influenced by the nature of mechanics‐driven energetic contributions to the forward and backward reaction rates. We hypothesize that different mechanics‐coupled reaction kinetics scenarios can translate into distinct electrodeposition stability regimes. To investigate this aspect, we extend the analysis performed in Figures 2, 3, 4 to other formulations listed in Table 1. **Figures** **5** and **6** present the stability maps for these formulations as a function of stack pressure, surface roughness, operating temperature and plating current density. Here, Case 1 ( $\delta_{\mathrm{mech}}^{\mathrm{Li}}=\delta_{\mathrm{mech}}^{Li^{+}}\;=\;0.5$) corresponds to the formulation where mechanics contributes equally to forward and backward reaction rates, and Case 2 ( $\delta_{\mathrm{mech}}^{Li^{+}}=\;1$ and $\delta_{mech}^{Li}=\;0$) and Case 3 ( $\delta_{\mathrm{mech}}^{Li^{+}}=\;0$ and $\delta_{mech}^{Li}=\;1$) correspond to the formulations where mechanics selectively contributes to only the forward and the backward reaction rate, respectively. We observe that Case 2 presents the narrowest stability regime (i.e., $i_{p}/i_{v}<1$), whereas Case 3 shows the widest stability regime. This difference in electrodeposition stability is related to the exchange current density and can be mechanistically explained as follows: In Case 1, the exchange current density, *i*_0_, is independent of the mechanical overpotential, $\eta_{\sigma }$ (see Table 1) and is therefore not affected by interfacial stresses (Figure 5d). The effect of mechanics on reaction kinetics is only through the equilibrium potential (Equation (10)). In Case 2, *i*_0_ is dependent on $\eta_{\sigma }$ as $i_{0}\propto \exp\left(\frac{F\eta_{\sigma }}{2RT}\right)$. Since $\eta_{\sigma }$ is more negative at the valleys, the magnitude of *i*_0_ will be lower in these regions as compared to that at the peaks (Figure 5e). As discussed in Figure 2, mechanical overpotential promotes stable electrodeposition by directing current to the valleys. However, this mechanism is countered by the lower exchange current density there, leading to lower reaction current at the valleys than in Case 1. As a result, the stability regime narrows, as observed in Figure 5b, and Figure 6b,e. In the third formulation (Case 3), *i*_0_ is dependent on $\eta_{\sigma }$ as $i_{0}\propto \exp\left(-\frac{F\eta_{\sigma }}{2RT}\right)$, which implies that the magnitude of exchange current density in this case will be greater at the valleys than at the peaks (Figure 5f). Thus, in addition to the stabilizing effect of mechanical overpotential, the conducive spatial distribution of exchange current density promotes a higher reaction current at the valley than in Case 1. This results in a broader stability regime for Case 3 (Figures 5c and 6c,f). In summary, despite the same interfacial stress profiles, the degree of mechanics‐driven stabilization achieved in Case 2 is the least, followed by Case 1, and maximum stabilization is achieved in Case 3.

Determining the exact degree of mechanics‐driven energetic contributions to the electrodeposition rate for different SE materials is crucial for achieving higher energy densities in SSBs by facilitating the creation of stable interfaces with the Li metal anode under desirable operating conditions. For instance, SE materials following the mechanics‐coupled kinetics formulation corresponding to Case 3 allow stable electrodeposition at lower stack pressures and operating temperatures than those following Case 1 or 2 (Figure 6a,b,c). Further, SE materials conforming to Cases 1 & 3 can support stable electrodeposition at high current densities, up to 3 mA/cm^2^, for moderate stack pressures (≈10 MPa), which can significantly boost the power density (Figures 6d,e,f). Therefore, thermodynamics‐informed selection and design of SE materials can lead to stable Li/SE interfaces, resulting in enhanced energy and power densities as well as longer cycle life and rate performance for SSBs.

**Figure 7:**
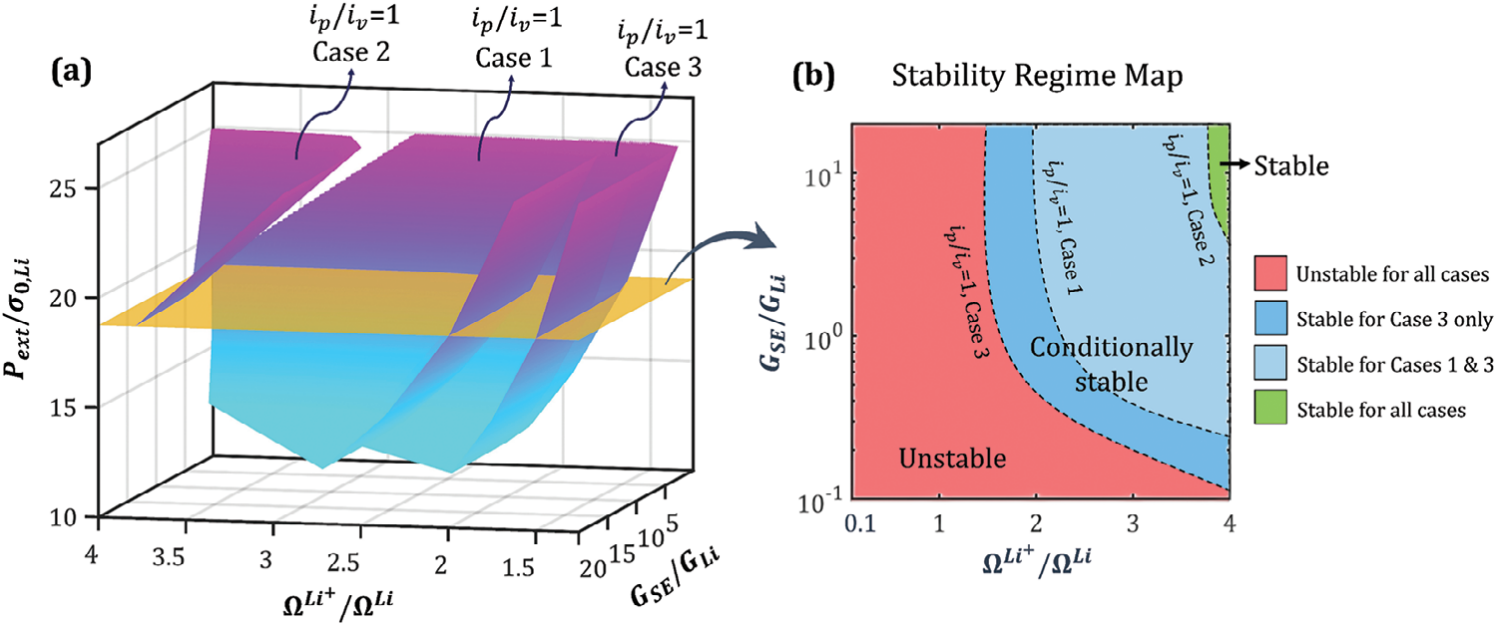
Electrodeposition stability landscape for different scenarios of mechanics‐driven energetic contributions to reaction kinetics as a function of mechanical and thermodynamic properties and stack pressure. a) *i_p_*/ *i_v_* = 1 isosurfaces as a function of the shear modulus ratio ($G_{\mathrm{SE}}/G_{\mathrm{Li}}$), partial molar volume ratio ($\Omega^{Li^{+}}/\Omega^{Li}$) and non‐dimensional stack pressure ($P_{\mathrm{ext}}/\sigma_{0,\mathrm{Li}}$) corresponding to Cases 1, 2, and 3 listed in Table 1. For each case, stable (unstable) electrodeposition is observed in the region above (below) the respective surface. b) Stability regime map at a stack pressure of 15 MPa across different mechanics‐coupled kinetics formulations listed in Table 1.

Electrodeposition stability regimes derived from mechanics‐driven energetic contributions to the reaction rate can be strongly dependent on the intrinsic thermodynamic and mechanical properties of Li metal and the SE. **Figure** **7** presents a consolidated electrodeposition stability map for different scenarios of mechanics‐reaction kinetics coupling as a function of the mechanical property descriptor $G_{\mathrm{SE}}/G_{\mathrm{Li}}$ (shear modulus ratio), thermodynamic property descriptor $\Omega^{Li^{+}}/\Omega^{Li}$ (partial molar volume ratio) and the non‐dimensional stack pressure. The latter is defined as the ratio of the stack pressure to the yield strength of Li metal ($P_{\mathrm{ext}}/\sigma_{0,\mathrm{Li}}$). Each surface in Figure 7a represents an $i_{p}/i_{v}=1$ isosurface corresponding to the mechanics‐coupled reaction kinetics formulations listed in Table 1. The region above/below the isosurface denotes the stable ($i_{p}/i_{v}<1$)/unstable ($i_{p}/i_{v}>1$) electrodeposition regime for the respective formulation. Figure 7b is a cross‐section of Figure 7a at a stack pressure of 15 MPa, depicting stability regimes across distinct mechanics‐coupled reaction kinetics scenarios as a function of the shear modulus ratio ($G_{\mathrm{SE}}/G_{\mathrm{Li}}$) and partial molar volume ratio ($\Omega^{Li^{+}}/\Omega^{Li}$). Based on our hypothesis that Li/SE interfaces can exhibit varying degrees of mechanics‐reaction kinetics coupling depending on the SE material, Figure 7b can be interpreted as follows. Consider a Li/SE interface under a stack pressure of 15 MPa, with properties of the SE in the region marked in red. Electrodeposition at this interface will always be unstable, regardless of the nature of mechanics contribution to the reaction rate. On the other hand, for a SE with properties in the green region, electrodeposition at the Li/SE interface will always be stable. Now consider two SEs with properties in the blue and pale blue regions, respectively. Electrodeposition stability for these two SEs is dependent on the nature of mechanics‐reaction kinetics coupling at the Li/SE interface. For the former, only Case 3 can yield stable electrodeposition, while for the latter, Cases 1 and 3 allow stable electrodeposition, but not Case 2. To sum it up, Cases 2 and 3 represent two extremes of the energetic contribution of mechanics to reaction kinetics, affecting only the rate of the forward and backward reaction, respectively. For any choice of SE material beyond the isosurfaces for Cases 2 and 3, electrodeposition at the Li/SE interface will be unconditionally stable (green in Figure 7b) and unstable (red in Figure 7b), respectively. The region bounded by the isosurfaces of Cases 2 and 3 (blue in Figure 7b) denotes the regime of conditional stability. In this regime, a precise understanding of the degree of mechanics contribution to reaction kinetics is necessary in order to ensure stable electrodeposition at the Li/SE interface.

The consolidated stability map illustrates that the inherent mismatch between the mechanical and thermodynamic properties of Li metal and the SE can give rise to distinct electrodeposition stability scenarios. Additionally, the stability regime is intricately influenced by operating conditions such as stack pressure and temperature. For SE materials whose degree of mechanics‐driven energetic contribution to the reaction rate is known, the consolidated stability map will indicate the stack pressure and temperature that will prevent the initiation of interfacial instabilities. For SEs where the degree of mechanics‐kinetics coupling cannot be determined accurately, the consolidated stability map suggests that the mechanics‐coupled reaction kinetics formulation corresponding to Case 2 can be used from a factor‐of‐safety perspective, since stable electrodeposition for Case 2 guarantees unconditional stability of the Li/SE interface. Additional considerations such as material heterogeneities within the SE, fracture mechanics of the SE and chemical stability of the SE with Li metal can hold further potential for refinement of the consolidated stability map.

A key highlight of our work is the investigation of distinct mechanics‐driven energetic contributions to the free energy landscape of the reaction and delineating its effect on the equilibrium potential and exchange current density. In this regard, a recent experimental study^[^ ^44^ ^]^ demonstrated that the equilibrium potential was proportional to the molar volume of the metal electrode, and the stress state of the single‐ion conducting SEs used in their study had negligible effect on the interfacial thermodynamic state. This corresponds to the Case 3 mechanics‐coupled kinetics scenario in our analysis. Carefully designed experiments are further required to identify the precise nature of mechanics‐driven energetic contributions to the reaction rate for different SE materials.

The results of our work are in line with experimentally observed trends for interface stability. For instance, an experimental study^[^ ^52^ ^]^ reported a marked increase in the critical current density (CCD) and a significant decrease in the Li‐ Li_7_La_3_Zr_2_O_12_ (LLZO) interfacial resistance as the operating temperature was increased from 30 to 175 °C. This is in accordance with our observation of increasing electrodeposition stability with temperature. Another study^[^ ^34^ ^]^ investigated the effect of stack pressure and reported an increase in interface area and a drop in interfacial resistance with increasing stack pressure for Li|Li_6_PS_5_Cl|Li cells. This is in line with our model results, which suggest increasing electrodeposition stability with stack pressure. However, several studies have also observed metal penetration in the SE at moderate to high stack pressures.^[^ ^34^, ^35^, ^36^ ^]^ Here, we would like to highlight that electrodeposition stability, as delineated in our work, corresponds to the initiation of interfacial instabilities that lead to Li filament growth. The stability regimes outlined in our analysis stem from the initial reaction distribution at the Li/SE interface triggered by a small sinusoidal interface perturbation. Within the unstable electrodeposition regime, material heterogeneities within the SE, such as grains, grain boundaries, and cracks will play a critical role in governing filament propagation. Although our analysis indicates that mechanical stresses can avert the onset of interfacial instabilities, these stresses should lie below the fracture threshold of the SE material to prevent mechanical failure caused by stack pressure. Thus, relying solely on stack pressure as a system control may not be the ultimate solution to enable stable electrodeposition. A fundamental understanding of the intricately coupled effect of operating conditions (including stack pressure and temperature) is critical toward achieving stable interfaces in SSBs.

Our primary focus in this manuscript lies in unraveling the thermodynamic foundations of mechanics‐coupled reaction kinetics and underscoring its fundamental role in the onset of interfacial instabilities by influencing the reaction distribution at the solid/solid interface. Building on the current framework, the propagation of these instabilities through crack formation and metal penetration in the SE, considering non‐conformal contact at the Li/SE interface, material heterogeneities within the SE, and the fracture mechanics of the SE will be studied in a future work.

## 4 Conclusion

This study investigates the thermodynamic underpinnings of the role of mechanics in reaction kinetics at the Li/SE interface and its ramifications on electrodeposition stability in SSBs. Using the transition state theory, we examine how mechanical stresses alter the free energy landscape of the redox reaction at the Li/SE interface and correlate it to changes in the equilibrium potential and exchange current density. Subsequently, we derive formulations for mechanics‐coupled reaction kinetics emerging from different possible mechanics‐driven energetic contributions to the forward (dissolution) and backward (deposition) reaction rates. Integrating this thermodynamic analysis into a coupled electro‐chemo‐mechanical model, we establish the competing effects of mechanical and electrical overpotentials on the reaction distribution at the interface. We show that interfacial stresses favor Li deposition in the valleys, facilitating stable electrodeposition which can be leveraged toward achieving stable interfaces in SSBs by modulating the intrinsic and extrinsic factors such as interface morphology, stack pressure, applied current density and operating temperature. We further reveal that distinct scenarios of mechanics‐reaction kinetics coupling lead to widely varying electrodeposition stability landscapes, and the smallest and largest stability regimes are obtained for scenarios where mechanics contributes only to the forward and backward reaction rates, respectively. Finally, we propose that Li/SE interfaces exhibit varying degrees of mechanics‐reaction kinetics coupling depending on the SE material and present a consolidated design map outlining conditional and unconditional stability regimes for diverse SE material properties. Overall, this work underscores the necessity of elucidating the precise role of mechanics in reaction kinetics for the rational design of stable Li/SE interfaces in SSBs.

## Conflict of Interest

The authors declare no conflict of interest.

## Supporting information

Supporting Information

## Data Availability

The data that support the findings of this study are available from the corresponding author upon reasonable request.
