# Supplementary material for: Electrodeposition Stability Landscape for Solid–Solid Interfaces
Source: Adv Sci (Weinh). 2023 Dec 10;11(6):2307455. doi: 10.1002/advs.202307455 (PMC10853722; doi:10.1002/advs.202307455)
Supplement: Supplementary file 1 — Supporting Information [file ADVS-11-2307455-s001.pdf]

ADVANCED SCIENCE
Open Access

Supporting Information

for *Adv. Sci.*, DOI 10.1002/advs.202307455

Electrodeposition Stability Landscape for Solid–Solid Interfaces

*Debanjali Chatterjee, Kaustubh G. Naik, Bairav S. Vishnugopi and Partha P. Mukherjee**

Supporting Information

Electrodeposition Stability Landscape for Solid-Solid Interfaces

Debanjali Chatterjee, Kaustubh G. Naik, Bairav S. Vishnugopi, and Partha P. Mukherjee[*]

School of Mechanical Engineering, Purdue University, West Lafayette, IN 47907, USA

[*]*Correspondence*: pmukherjee@purdue.edu

S1. Modeling framework & methodology:

The computational domain comprises of Li metal and SE under fixed stack pressure (P_{ext}) and applied current density (i_{app}). Li metal is considered to be elastic-perfectly plastic, whereas the SE is elastic. Owing to solid-solid contact between rough surfaces, the Li metal-SE interface is perturbed, giving rise to non-uniform interfacial stresses under stack pressure (Figure S1). The interface is modeled as a sinusoidal function $y = A\sin(\omega x)$, where A and ω denote the surface roughness amplitude and frequency, respectively. The stress field in the domain can be obtained by solving the quasistatic equilibrium equation in a plane strain configuration[1]:

$$\vec{\nabla}.\overleftrightarrow{\sigma} = 0 \tag{S1}$$

Where $\vec{\nabla}$ denotes the gradient operator and $\overleftrightarrow{\sigma}$ indicates the stress tensor. Linear elastic mechanics in the SE gives the stress field in the SE domain as follows:

$$\overleftrightarrow{\epsilon} = \frac{1}{2}(\nabla\vec{u} + (\nabla\vec{u})^{\mathrm{T}}) \tag{S2}$$

$$\overleftrightarrow{\sigma} = 2\mu\overleftrightarrow{\epsilon} + \lambda\,\mathrm{tr}(\overleftrightarrow{\epsilon})I \tag{S3}$$

Where μ and λ are the Lamé constants given by $\mu = \frac{E_{SE}}{2(1+\nu_{SE})}$ and $\lambda = \frac{E_{SE}\nu_{SE}}{(1+\nu_{SE})(1-\nu_{SE})}$, E_{SE} and ν_{SE} being the Young's modulus and Poisson's ratio of the SE, respectively. tr is the trace operator and I is the identity matrix. $\vec{u}$ and $\overleftrightarrow{\epsilon}$ denote the displacement field and the strain tensor, respectively. Stresses in the Li domain arising from the plastic deformation of Li metal have been incorporated as prescribed by Barai et. al[2].

Li^+ ion transport in the SE is governed by the charge conservation equation:

$$\nabla.(\kappa \nabla \phi^{SE}) = 0 \tag{S4}$$

where ϕ^{SE} is the electric potential (V) in the SE and κ is its effective ionic conductivity (S/m). Inorganic SEs are considered to be single-ion conductors owing to their high transference number (~1). Therefore, concentration gradients within the SE are negligible.

Electron transport in Li metal is governed by the following equation:

$$\nabla.\left(\sigma \nabla \phi^{Li}\right) = 0 \tag{S5}$$

where ϕ^{Li} is the solid-phase potential of Li metal and σ is its electronic conductivity (S/m). Since Li metal is highly conductive[2], variation in ϕ^{Li} is negligible and it is considered to be zero throughout the Li domain.

The boundary conditions for mechanical equilibrium (Equation S1) and charge conservation in the SE (Equation S4) are listed in Table S1.

Electrodeposition at the Li metal-SE interface follows Butler-Volmer kinetics (Equation 12). The equilibrium potential of Li deposition at standard temperature and pressure, E^0, is considered to be 0 V. This gives the surface overpotential (Equation 13):

$$\eta = \eta_e + \eta_\sigma = -\phi^{SE} + \eta_\sigma \quad \text{(S6)}$$

First, the mechanical overpotential (η_σ) is computed from interfacial hydrostatic stresses (Equation 10). This is followed by iteratively solving for the electric potential in the SE (ϕ^{SE}) (Equation S4) using the mechanics-coupled reaction kinetics formulation (Table 1) as the boundary condition at the Li/SE interface. Once the mechanical (η_σ) and electrical (η_e) overpotentials are calculated, the reaction distribution at the interface can be obtained. The electrical overpotential at the Li/SE interface is strongly influenced by the mechanical overpotential (η_σ) through the mechanics dependence of reaction kinetics, resulting in an intricate electro-chemo-mechanical coupling.

The ionic conductivity of the SE, κ, and mechanics-independent exchange current density, i_{00}, exhibit a strong dependence on temperature as given by the Arrhenius equation[1]:

$$\kappa = \kappa_{ref} \exp\left(-\frac{E_{a,\kappa}}{R}\left(\frac{1}{T} - \frac{1}{T_{ref}}\right)\right) \quad \text{(S7)}$$

$$i_{00} = i_{00,ref} \exp\left(-\frac{E_{a,i_{00}}}{R}\left(\frac{1}{T} - \frac{1}{T_{ref}}\right)\right) \quad \text{(S8)}$$

Where T is the operating cell temperature, $E_{a,\kappa}$ and $E_{a,i_{00}}$ are the activation energies of the SE ionic conductivity and mechanics-independent exchange current density, respectively, and κ_{ref} and $i_{00,ref}$ are the respective values of the SE ionic conductivity and the mechanics-independent exchange current density at the reference temperature, T_{ref} = 25°C. It must be noted that the activation energies of the forward and backward reactions do not change with temperature. However, at higher temperatures, a greater proportion of Li atoms / Li^+ ions have the kinetic energy to overcome the forward / backward activation barriers, which results in an increase in

the corresponding reaction rates ($r_f = \frac{k_B T}{h} \exp\left(-\frac{E_{a,f}}{RT}\right)$ and $r_b = \frac{k_B T}{h} \exp\left(-\frac{E_{a,b}}{RT}\right)$). This translates into higher exchange current density with increasing temperature, as described in Equation S8. While elevated temperatures lead to an overall increase in the reaction rate across the entire Li/SE interface, the spatial homogenization of reaction current is primarily attributed to

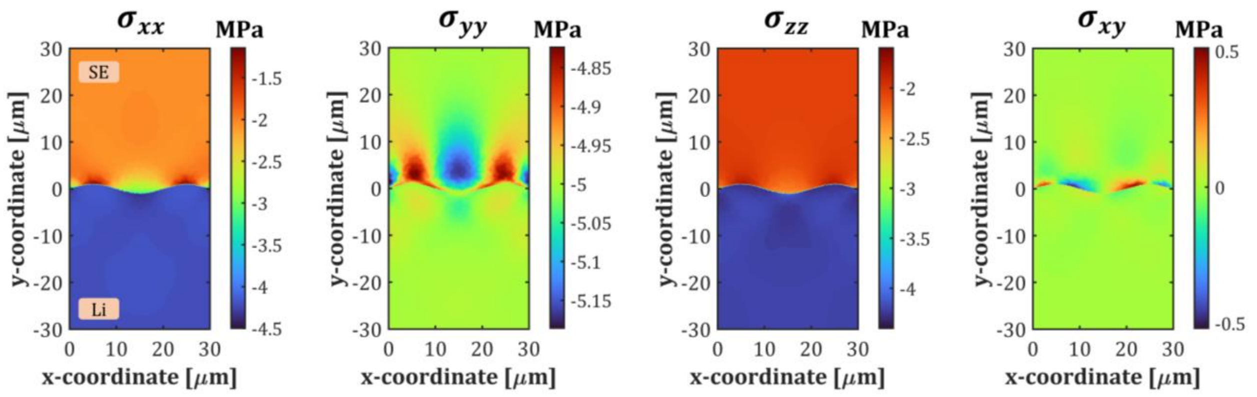

the uniform electric potential gradients near the interface due to efficient ionic transport in the SE. Temperature also influences the contribution of mechanical stresses to reaction kinetics, and hence, it is intricately coupled to the electrochemical and mechanical driving forces that govern lithium deposition at the interface.

All the parameters and their values used in the modeling framework are listed in Table S2.

Figure S1. Stress components in the Li-SE domain under a stack pressure of 5 MPa.

Table S1: Boundary conditions for the Li-SE domain in SSB under stack pressure.

**Domain**	**Boundary**	**Mechanics** $\vec{\nabla}.\overleftrightarrow{\sigma} = 0$	**Li^+ ion transport** $\nabla.(\kappa\nabla\phi^{SE}) = 0$
SE	Top surface ($y = H_{SE}$)	$\overleftrightarrow{\sigma}\vec{n} = P_{ext}\,\vec{n}$	$-\kappa\nabla\phi^{SE} = i_{app}$
	Li-SE interface ($y = A\,sin(\omega x)$)	-	$-\kappa\nabla\phi^{SE} = i_{BV}$
	Lateral faces ($x = 0, W$)	$u_x = 0, \frac{\partial u_y}{\partial x} = 0$	$-\kappa\nabla\phi^{SE} = 0$
Li	Bottom surface ($y = -H_{Li}$)	$u_x, u_y = 0$	-
	Lateral faces ($x = 0, W$)	$u_x = 0, \frac{\partial u_y}{\partial x} = 0$	-

Table S2: List of properties and parameters used in the modeling framework.

Parameters		Values	Units	Reference
E_{Li}	Young's modulus of Li	7.82	GPa	[3]
G_{Li}	Shear modulus of Li	2.83	GPa	[3]
ν_{Li}	Poisson's ratio of Li	0.381	-	[3]
$\sigma_{0,Li}$	Yield strength of Li	0.8	MPa	[3]
E_{SE}	Young's modulus of SE	11	GPa	[1]
ν_{SE}	Poisson's ratio of SE	0.29	-	[1]
Ω^{Li}	Partial molar volume of Li	1.3×10^{-5}	$m^3\ mol^{-1}$	[2]
Ω^{Li^+}	Partial molar volume of Li^+ ion in SE	$5\Omega^{Li}$ (unless specified otherwise)	$m^3\ mol^{-1}$	[4]
κ_{ref}	Effective ionic conductivity of SE at reference temperature	0.03	$S\ m^{-1}$	[1]
σ	Electronic conductivity of Li	1.1×10^7	$S\ m^{-1}$	[2]
$i_{00,ref}$	Mechanics-independent exchange current density	0.781	$mA\ cm^{-2}$	[1]
$E_{a,\kappa}$	Activation energy for ion transport in SE	24.04	$kJ\ mol^{-1}$	[1]
$E_{a,i_{00}}$	Activation energy for charge transfer	48.54	$kJ\ mol^{-1}$	[1]
F	Faraday's constant	96485.33	$C\ mol^{-1}$	-
R	Universal gas constant	8.314	$J\ mol^{-1}K^{-1}$	-
T	Operating temperature	298.15 (unless specified otherwise)	K	-
T_{ref}	Reference temperature	298.15	K	-
W	Domain width	30	μm	-
H_{Li}	Thickness of Li metal anode	50	μm	-
H_{SE}	Thickness of SE	50	μm	-
A	Surface roughness amplitude	1 (unless specified	μm	-

		otherwise)		
ω	Surface roughness frequency	$3\pi/W$	m^{-1}	-

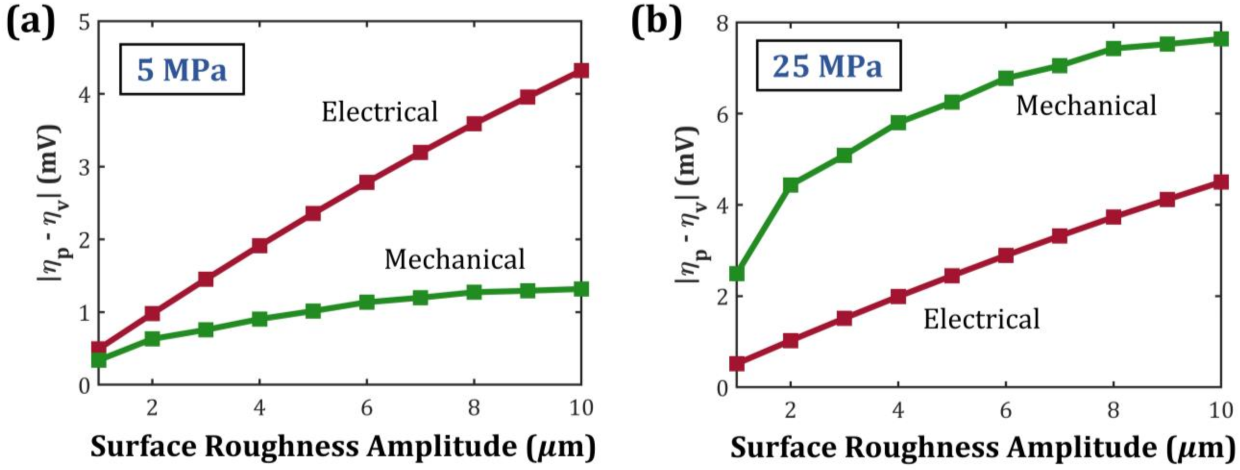

Figure S2. Difference between overpotentials at the interface peak and valley, for both mechanical and electrical overpotentials, at stack pressures of (a) 5 MPa and (b) 25 MPa.

References:

(1) Verma, A.; Kawakami, H.; Wada, H.; Hirowatari, A.; Ikeda, N.; Mizuno, Y.; Kotaka, T.; Aotani, K.; Tabuchi, Y.; Mukherjee, P. P. Microstructure and pressure-driven electrodeposition stability in solid-state batteries. *Cell Reports Physical Science* **2021**, *2* (1), 100301.

(2) Barai, P.; Higa, K.; Srinivasan, V. Lithium dendrite growth mechanisms in polymer electrolytes and prevention strategies. *Physical Chemistry Chemical Physics* **2017**, *19* (31), 20493-20505.

(3) Masias, A.; Felten, N.; Garcia-Mendez, R.; Wolfenstine, J.; Sakamoto, J. Elastic, plastic, and creep mechanical properties of lithium metal. *Journal of materials science* **2019**, *54* (3), 2585-2600.

(4) Mistry, A.; Mukherjee, P. P. Molar volume mismatch: a malefactor for irregular metallic electrodeposition with solid electrolytes. *Journal of the Electrochemical Society* **2020**, *167* (8), 082510.
